# A Model of the Spatio-temporal Dynamics of *Drosophila* Eye Disc Development

**DOI:** 10.1371/journal.pcbi.1005052

**Published:** 2016-09-14

**Authors:** Patrick Fried, Máximo Sánchez-Aragón, Daniel Aguilar-Hidalgo, Birgitta Lehtinen, Fernando Casares, Dagmar Iber

**Affiliations:** 1 Department of Biosystems, Science and Engineering (D-BSSE), ETH Zurich, Basel, Switzerland; 2 Swiss Institute of Bioinformatics (SIB), Basel, Switzerland; 3 CABD, CSIC and Universidad Pablo de Olavide, Campus UPO, Seville, Spain; Ecole Normale Supérieure, FRANCE

## Abstract

Patterning and growth are linked during early development and have to be tightly controlled to result in a functional tissue or organ. During the development of the *Drosophila* eye, this linkage is particularly clear: the growth of the eye primordium mainly results from proliferating cells ahead of the morphogenetic furrow (MF), a moving signaling wave that sweeps across the tissue from the posterior to the anterior side, that induces proliferating cells anterior to it to differentiate and become cell cycle quiescent in its wake. Therefore, final eye disc size depends on the proliferation rate of undifferentiated cells and on the speed with which the MF sweeps across the eye disc. We developed a spatio-temporal model of the growing eye disc based on the regulatory interactions controlled by the signals Decapentaplegic (Dpp), Hedgehog (Hh) and the transcription factor Homothorax (Hth) and explored how the signaling patterns affect the movement of the MF and impact on eye disc growth. We used published and new quantitative data to parameterize the model. In particular, two crucial parameter values, the degradation rate of Hth and the diffusion coefficient of Hh, were measured. The model is able to reproduce the linear movement of the MF and the termination of growth of the primordium. We further show that the model can explain several mutant phenotypes, but fails to reproduce the previously observed scaling of the Dpp gradient in the anterior compartment.

## Author Summary

Patterning and growth of a tissue are linked during early development and have to be tightly controlled. During the development of the *Drosophila* eye, this linkage is particularly clear: A moving signaling wave sweeps across the tissue that will eventually develop into the eye of the fly. This wave is responsible for the transition from cells undergoing cell divisions in front of the wave into differentiated, specialized cells that are not dividing anymore and that eventually develop into the many individual eye units of the compound eye. Therefore, the final size of this tissue depends on how fast cells in front of the wave are growing and dividing and on the speed with which the signaling wave sweeps across the tissue. We developed a computational model based on regulatory interactions that have been experimentally determined in order to explore how the signaling patterns affect the movement of the signaling wave and impact on tissue growth. The model captures the movement of the signaling wave at a constant speed and the growth termination of the developing tissue. We further show that the model can explain the abnormal size of the eye that can be observed in several genetically modified fly strains.

## Introduction

**Fig 1:**
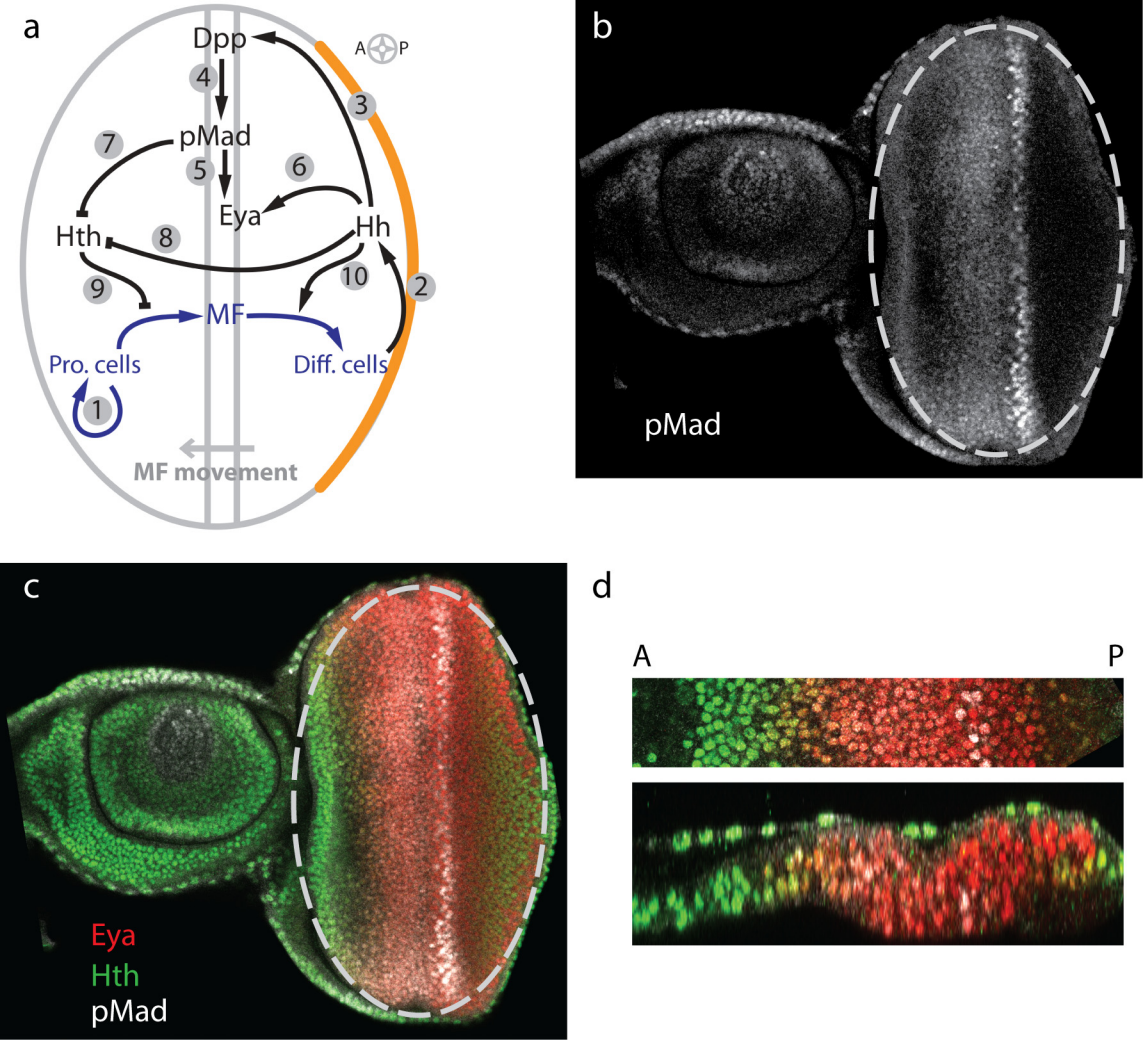
Model of regulatory network during eye disc development. (a) During eye disc development the MF is initiated at the posterior margin and sweeps across the eye disc in an anterior direction. In front of the MF progenitor cells proliferate (arrow (A)1), while behind the MF cells differentiate and eventually form the ommatidia. Hh is expressed in the posterior margin (marked in orange), from where it diffuses into the eye disc (A2), and initiates expression of *dpp* in the MF (A3). Dpp signals by phosphorylation of Mad to pMad (A4). pMad (A5) as well as Hh (A6) enhance the expression of *eya*. Both pMad-mediated Dpp signaling (A7) as well as Hh signaling (A8) repress the expression of *hth*. Initially Hth is present throughout the disc. As the Hth levels decline, progenitor cells can transit into MF cells (A9). Hh supports the differentiation process by inducing the differentiation of MF cells (A10). Thus, Hh and Dpp/pMad together drive the progression of the MF. (b,c) A representative single (x,y) confocal section of a late third instar eye-antennal disc with only pMad staining (b) or merged channels (Hth: green, Eya: red; PMad: white) (c). The eye primordium is marked with an ellipse in (b,c). (d) A magnified sector of a similar eye disc along the A-P axis (top) and a z-section through this sector as in (bottom). Same color code as in (c).

During early development most tissues undergo fast changes involving cell growth, proliferation, differentiation and patterning. In order to develop into a functional tissue or organ, both patterning and growth have to be tightly controlled and coordinated. How this regulation is achieved is an extraordinarily complex problem. As is the case with many fundamental mechanisms, also the interplay between growth and patterning has been most widely investigated in *Drosophila* [1–4]. One tissue of particular interest in *Drosophila* is the eye imaginal primordium (commonly called eye “imaginal disc”), that develops into the highly organized compound eye of adult flies. During the two first larval stages (or “instars”) the eye disc comprises undifferentiated, proliferative eye progenitors. Pattern formation in the eye disc starts during early third instar with the appearance of the morphogenetic furrow (MF), a straight epithelial indentation that runs along the dorsoventral (DV) axis of the *Drosophila* eye disc and that emerges at the posterior margin of the disc [5]. The MF is a moving signaling centre that separates the proliferating (anterior to the MF) and the differentiating (posterior to the MF) zones in the disc (Fig 1A). As the MF sweeps across the disc from its posterior to its anterior side, undifferentiated proliferating cells cease to proliferate at the MF and start differentiating into retina cells behind it [5]. The velocity of MF movement thus determines the time for which cells on the anterior side of the disc can proliferate.

At a molecular level, many signals that are involved in the initiation and progression of the morphogenetic furrow have been described [6]. Initiation of MF movement requires the production of the diffusible morphogen Hedgehog (Hh) at the posterior eye disc margin. Hh is known to induce the expression of *decapentaplegic* (*dpp*) in the MF, another morphogen of the BMP2/4 type, and to promote differentiation of the proliferating cells into retinal cells. Furthermore, it is known that both Hh and Dpp signaling lead to the downregulation of the transcription factor *homothorax* (*hth*) [7]. *hth* is expressed on the anterior side of the eye disc and was shown to promote cell proliferation and to block the expression of later-acting transcription factors [7,8]. Behind the MF, the newly differentiated photoreceptor cells express *hh* and the delayed mutual activating loop between Hh and Dpp is able to set the MF in motion. Recent measurements describe this movement of the MF as linear in time [9]. However, it remains unclear how the signaling network determines the speed of the MF. By driving the progression of the MF, the signaling network is indirectly regulating proliferation.

In addition to driving MF movement and cell differentiation, Dpp signaling affects growth [10–12]. Recently, it was reported that the gradients of the Dpp signaling targets pMad and *hairy* scale with the anterior length of the eye disc, and the authors suggested that the relative temporal changes in the concentration of the moving Dpp gradient control the proliferation rate and are therefore responsible for the control of growth and its termination [9]. However, the authors also note that the growth rate is not altered when the only Dpp signalling mediator *mad* and its downstream target *brk* are removed from cells.

In order to explore how the spatio-temporal signaling patterns affect the movement of the MF and impact on eye disc growth we translated the signaling network into a spatio-temporal model (Fig 1A). Patterning and growth are intricately linked during eye disc development, and we therefore solved the model on a growing domain. As in previous models of imaginal disc growth [13], we modeled the epithelium as an incompressible Newtonian fluid with a source that reflects cell proliferation. In order to parameterize the model we measured two key parameters of the model, the degradation rate of Hth as well as the diffusion coefficient of Hh, by Fluorescent Recovery After Photobleaching (FRAP). We show that our model can reproduce the linear movement and speed of the MF. Our model shows the observed growth termination and can reproduce several mutant phenotypes that influence the speed of the MF. We furthermore analyze the impact of parameter perturbations on the linearity and speed of the MF as well as on the final size of the eye disc. Importantly, the model fails to reproduce the scaling of the Dpp gradient with the anterior length of the tissue, suggesting that there must be additional mechanisms in place to ensure scaling. While many open questions remain, our model serves as an important step towards an integrated model for patterning and growth control during development.

## Results

### Development of the Eye Disc Model

We aimed at developing a parsimonious model for eye disc growth and early patterning and thus sought to keep the regulatory interactions as simple as possible while reproducing the measurements. As components of the model we will consider Hh, Dpp, pMad (the active form of Mad which transduces the Dpp signal to the nucleus [14]), Eya, a gene expressed and required for retinal specification and differentiation [15] and Hth, a protein that prevents premature differentiation anterior to the MF (Fig 1A). Representative confocal sections of similar late third instar eye-antennal discs stained for several proteins that are incorporated in the model are shown from different views in Fig 1B–1D. We will focus on the differentiation process beginning with the initiation of the morphogenetic furrow (MF) in larvae during early third instar. In front of the MF, progenitor cells proliferate (Fig 1A; arrow (**A**) **1**), while behind the MF cells differentiate and eventually form the ommatidia. Experiments show that *hh* is expressed at the posterior margin before MF initiation and in differentiated cells, labeled Φ, posterior to the MF during disc development [16] (Fig 1A; **A2**). The production in the margin is incorporated via the boundary condition for Hh (Eq 19, see Methods). Given the lack of evidence for any relevant regulation of the production of *hh* by other members in the model, we assume a constant production rate, restricted to the differentiated cells, thus writing *p*_*Hh*_ ⋅ Φ. Hh induces the expression of *dpp* in MF cells, labeled Θ [17] (Fig 1A; **A3**). In order to keep the number of parameters small, we use the simplest function that allows us to fit the data, a linear relationship. Accordingly we write *p*_*Dpp*_ ⋅ *c*_*Hh*_ ⋅ Θ as a production term, with *c*_*Hh*_ being Hh concentration. We note that also a Hill function would have allowed us to reproduce the observed data. However, this would have introduced two additional parameters. Dpp signaling is mediated by phosphorylation of Mad to pMad [14] (Fig 1A; **A4**). The rate of Mad phosphorylation thus depends on the Dpp concentration, and we have *p*_*pMad*_ ⋅ *σ*_*Dpp*_ as a production term for pMad (see definition for *σ* in Eq 3). Expression of *eya* is enhanced by pMad-mediated Dpp signaling [18] (Fig 1A; **A5**). As the expression of *eya* is also induced by Hh [19] we incorporated a Hh-dependent term (Fig 1A; **A6**) and a pMad-dependent production term, such that the presence of either pMad or Hh is sufficient to induce *eya* expression, i.e. *p*_*Eya*_ ⋅ (*σ*_*pMad*_ + *σ*_*Hh*_). We notice that we could also have reproduced the mutant behavior if we substituted the Hh-dependent term by a positive feedback of eya on itself or by a direct link of the photoreceptor cells. *hth* can be expressed in all cells, but is repressed by pMad-mediated Dpp signaling (Fig 1A; **A4**, **A7**), and by Hh signaling (Fig 1A; **A8**) [20,21]. We therefore describe Hth production by $p_{Hth}\cdot {\bar{\sigma }}_{pMad}\cdot {\bar{\sigma }}_{Hh}$, such that the presence of either pMad or Hh is sufficient to repress *hth* expression (compare Eq 3). Hth is required to maintain the progenitor population in a proliferative and undifferentiated state (Fig 1A; **A9**) [7], while Hh is required for the proper differentiation of cells behind the MF into photoreceptor cells (Fig 1A; **A10**). Furthermore, forced maintenance of Hth is known to cause severe delays in MF movement and blocks retinal differentiation [22,23]. Downregulation of *hth* expression therefore allows MF movement [20,22,23]. The simplest way to incorporate these known concentration-dependent cell type transitions is to introduce an Hth concentration threshold, Θ_*Hth*_, below which proliferating cells are becoming MF cells and an Hh concentration threshold, Θ_*Hth*_, above which MF cells are differentiating into differentiated cells (see also Eq 4).

All extracellular molecules can diffuse within the domain, albeit at different speeds. We therefore formulate the model as advection-reaction-dispersion equations for a component *i* with concentration *c*_*i*_, diffusion coefficient *D*_*i*_ and reaction terms *R*_*i*_. The external velocity field is denoted by *u*:

$$\frac{\partial c_{i}}{\partial t}+\nabla (uc_{i})=D_{i}\nabla^{2}c_{i}+R_{i}$$ (1)

The reaction terms *R*_*i*_ of the components describe the regulatory interactions based on information from the literature and our own experiments as discussed above, and are given by:

$$R_{Hh}=p_{Hh}\cdot \Phi -\delta_{Hh}\cdot c_{Hh}$$ (2)

$$R_{Dpp}=p_{Dpp}\cdot c_{Hh}\cdot \Theta -\delta_{Dpp}\cdot c_{Dpp}$$

$$R_{pMad}=p_{pMad}\cdot \sigma_{Dpp}-\delta_{pMad}\cdot c_{pMad}$$

$$R_{Eya}=p_{Eya}\cdot (\sigma_{pMad}+\sigma_{Hh})-\delta_{Eya}\cdot c_{Eya}$$

$$R_{Hth}=p_{Hth}\cdot {\bar{\sigma }}_{pMad}\cdot {\bar{\sigma }}_{Hh}-\delta_{Hth}\cdot c_{Hth}$$

In the absence of contrary data we use the simplest model for decay, linear decay at rate *δ*_*i*_ ⋅ *c*_*i*_ for all signaling factors *i*. We use Hill functions to describe regulatory influences. To describe activating influences of a component *i* we write

$$\sigma_{i}=\frac{c_{i}^{n_{i}}}{c_{i}^{n_{i}}+K_{i}^{n_{i}}}$$ (3)

and we use ${\bar{\sigma }}_{i}=1-\sigma_{i}$ to describe inhibitory impacts of *c*_*i*_. *K*_*i*_ is the Hill constant which specifies the concentration of *c*_*i*_ where half-maximal activity is observed, and the Hill coefficient *n*_*i*_ defines the steepness of the response. The different cell types, i.e. differentiated cells Φ, cells in the MF Θ and proliferating cells Π, are defined as

$$\Pi =H(c_{Hth}-\theta_{Hth})$$ (4)

$$\Theta =(1-H(c_{Hth}-\theta_{Hth}))\cdot (1-H(c_{Hh}-\theta_{Hh}))$$

$$\Phi =(1-H(c_{Hth}-\theta_{Hth}))\cdot H(c_{Hh}-\theta_{Hh})$$

where *H*(*c*_*i*_ − *θ*_*i*_) for component *i* and threshold *θ*_*i*_ is the Heaviside function, which is defined according to:

$$H(c_{i}-\theta_{i})=\left\{ \begin{array}{c} \begin{array}{ccc} 0 & \text{if} & c_{i}\leq \theta_{i} \end{array}\\ \begin{array}{ccc} 1 & \text{if} & c_{i}>\theta_{i} \end{array} \end{array}\right.$$ (5)

Finally, we need to define the boundary and initial conditions. Hh is expressed in the margin from where it diffuses into the eye disc. Accordingly, we use as boundary condition for Hh

$$D_{Hh}\nabla c_{Hh}=\eta \cdot \Lambda (x)\cdot \tau (t)$$ (6)

where *η* is a constant, Λ(*x*) defines the spatial location of the Hh producing margin and *τ*(*t*) a time-dependent function (see Methods for details). We use zero flux boundary conditions for all other signaling molecules, transcription factors and cell types, i.e.

$$\nabla c_{i}=0$$ (7)

We use zero initial conditions for Hh, Dpp, pMad, and Eya in the entire eye disc domain; MF initiation happens in response to the influx of Hh at the posterior margin (see Methods). Before the initiation of the MF, *hth* is expressed in all cells of the eye primordium [23]. We therefore use the steady state concentration as initial concentration, i.e. $c_{Hth}(0)=\frac{p}{\delta_{Hth}}$. The presence of Hth prevents premature cell differentiation. In summary, the initial conditions are:

$$c_{Hth}(0)=\frac{p}{\delta_{Hth}}$$ (8)

$$c_{Hh}(0)=c_{Dpp}(0)=c_{pMad}(0)=c_{Eya}(0)=0$$

As previously established, the eye disc can be approximated by a 2D ellipse (Fig 1B and 1C) [24]. On long time scales, embryonic tissue can often be described by a viscous fluid [25,26]. Accordingly, we model the mechanical behaviour of the eye disc as an incompressible Newtonian fluid with density *ρ*, dynamic viscosity *μ* and local source *S*. This approach has previously been used in simulations of early vertebrate limb development [27] and, in an extended anisotropic formulation, to *Drosophila* imaginal disc development [13]. The Navier-Stokes equation is given as:

$$\rho \left(\frac{\partial u}{\partial t}+(\nabla \cdot u)u\right)=-\nabla \rho +\mu \left(\begin{array}{c} \nabla^{2}u+\frac{1}{3}\nabla (\nabla \cdot u) \end{array}\right)$$ (9)

$$\nabla \cdot u=S=\Pi \cdot k_{0}\cdot exp(-\delta_{PL}\cdot PL)$$

where *u* denotes the external velocity field used in Eq 1, *S*, from definition above, denotes the local growth rate and *PL* denotes the "posterior length", i.e. the length from the posterior margin to the MF (corresponding to the differentiating region) which is a good surrogate of developmental time given the linear progression of the MF with time [9,24]. *k*_0_ is the initial area growth rate and has been previously estimated from experimental data [24]. We assume that growth is caused by proliferation of undifferentiated cells only (Fig 1A; **A1**) and we have previously found that the measured growth rate can be described well by a function that declines exponentially with *PL*, with δ_*PL*_ = 0.0107 μm^-1^ [24]. Different mechanisms could, in principle, give rise to this measured decline [24]. However, in the absence of a confirmed growth-controlling mechanism, we decided to use the functional relation that most accurately describes the growth dynamics in the Drosophila eye disc [24] without making any statement about how growth may be regulated mechanistically. We also assume the same growth rule in both x and y directions. This is in agreement with experimental observations: the growth anisotropy parameter was previously determined as $\epsilon =\frac{\partial_{y}u}{\partial_{x}u}\approx 1$ [9].

### Parameterization of the Model and Determination of Missing Parameters

In most mathematical models the parametrization is crucial for its capabilities to correctly reflect the modeled phenomena. In our model we have three classes of parameters: Production rates and coefficients of the Hill terms, diffusion coefficients and degradation rates. As the absolute protein concentrations are unknown, the production rates can be set to arbitrary values and the Hill coefficients must then be adjusted to reproduce the experimentally observed protein gradients and gene expression boundaries. We used quantitative confocal microscopy of stained eye discs to detect the spatio-temporal dynamics of the core proteins pMad, Eya, and Hth. Fig 1B–1D shows representative confocal sections of a late third instar eye-antennal disc. For profile quantification, z-stacks of disc strips were acquired. A single (x,y) confocal section of one of these strips is shown (Fig 1B and 1C). Fig 1D shows a magnified section of a similar eye disc (top) and a z-section through that section (bottom). pMad or merged channels (Hth: green, Eya: red; pMad: white) are shown. As the Dpp-producing MF moves, the concentration profiles of pMad, Eya, and Hth also move towards the anterior side. For easier comparison of the concentration profiles we plot these relative to the MF (S1A–S1C Fig). The regulatory concentration thresholds could be adjusted such that the model reproduced the shapes of the concentration profiles of pMad, Eya, and Hth (S1A–S1C Fig, red lines). In this way, all production rates and Hill coefficients could be determined (S1 Table). With respect to the diffusion coefficients, we note that Hth, Eya, and pMad are intracellular proteins and therefore their diffusion across the tissue is negligible. In the simulations we could reflect this by setting *D* = 0 for all three species. However, we opted for a very low effective diffusion coefficient for numerical stability (*D* = 0.00025 μm^2^ s^-1^), which may also reflect intracellular diffusion. Regarding Dpp kinetic parameters, there are no experimental measures performed in the eye disc. However, in the wing disc, different groups have measured distinct properties regarding Dpp transport, reporting values for Dpp free extracellular diffusion [28], effective diffusion coefficient [28,29] and the length of the Dpp gradient [29]. As our model considers effective parameters we choose for the Dpp diffusion constant *D*_*Dpp*_ = 0.1 μm^2^ s^-1^ and, based on the Dpp gradient length of the wing disc, deduced the Dpp degradation rate as δ_*Dpp*_ = 2.5 × 10^−4^ s^-1^ [29]. This Dpp decay rate would correspond to a half-life of 45 min. We note that subsequent measurements showed that the Dpp gradient lengthens over time in the wing disc [4]. Based on this observation, it has been proposed that the Dpp degradation rate would decline over time [4]. However, we have since shown that the data can be fully explained with a constant degradation rate if the dynamic pre-steady state nature of the patterning process is taken into account [30]. While we estimated the Dpp half-life to be longer than 10 hours in the wing imaginal disc [30], we are here focused on the Dpp removal rate from the extracellular space, and the rate of Dpp internalization is fast. Therefore, we will use as degradation constant δ_*Dpp*_ = 0.00025 s^-1^.

**Fig 2:**
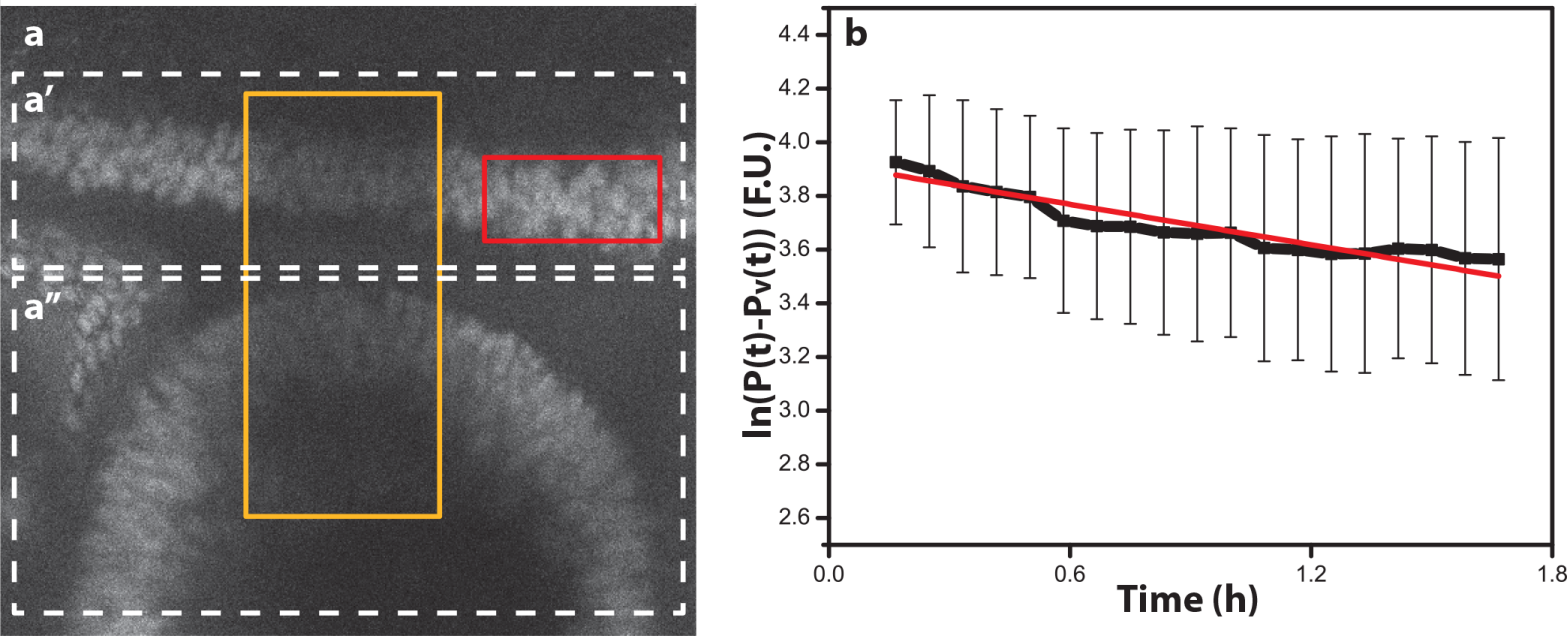
Determination of the Hth degradation rate. (a) View of the eye/antenna disc fragment. The recovery rate that was obtained on the antenna side (Fig 2a'') was within the standard deviation of the recovery rate obtained on the eye side (Fig 2a'). This implies that the degradation rate does not differ between these two tissues. (b) Time series of the logarithm of the relative Hth intensity between unbleached (red rectangle in Fig 2A) and bleached (yellow rectangle in Fig 2A) regions. The slope of the curve represents the degradation rate, *δ*_*Hth*_ = (6.97 ± 5.00) 10^−5^ s^-1^; the linear fit is shown as red line; R^2^ = 0.89.

Three crucial parameters have not been previously measured: the diffusion coefficient and the degradation rate of Hh and the degradation rate of Hth. Since the characteristic length of the Hh gradient has been determined, we focused on measuring the effective Hh diffusion coefficient and the Hth degradation rate. Both experiments were performed using FRAP and were obtained in the wing disc, as this disc is larger and flatter than the eye disc, thus facilitating the experiments and assuming the same dynamics in both disc types. Fig 2 shows the FRAP experiment for determining the Hth degradation rate. The degradation rate can be calculated by linearly fitting the time series for the bleach-chase analysis of Hth (Fig 2B) [31]. The slope of the fitted line yields the degradation rate, δ_*Hth*_ = (6.97 ± 5.00) 10^−5^ s^-1^, which corresponds to a Hth protein half-life of τ_1/2_ = 2.77 h (see details in Materials).

**Fig 3:**
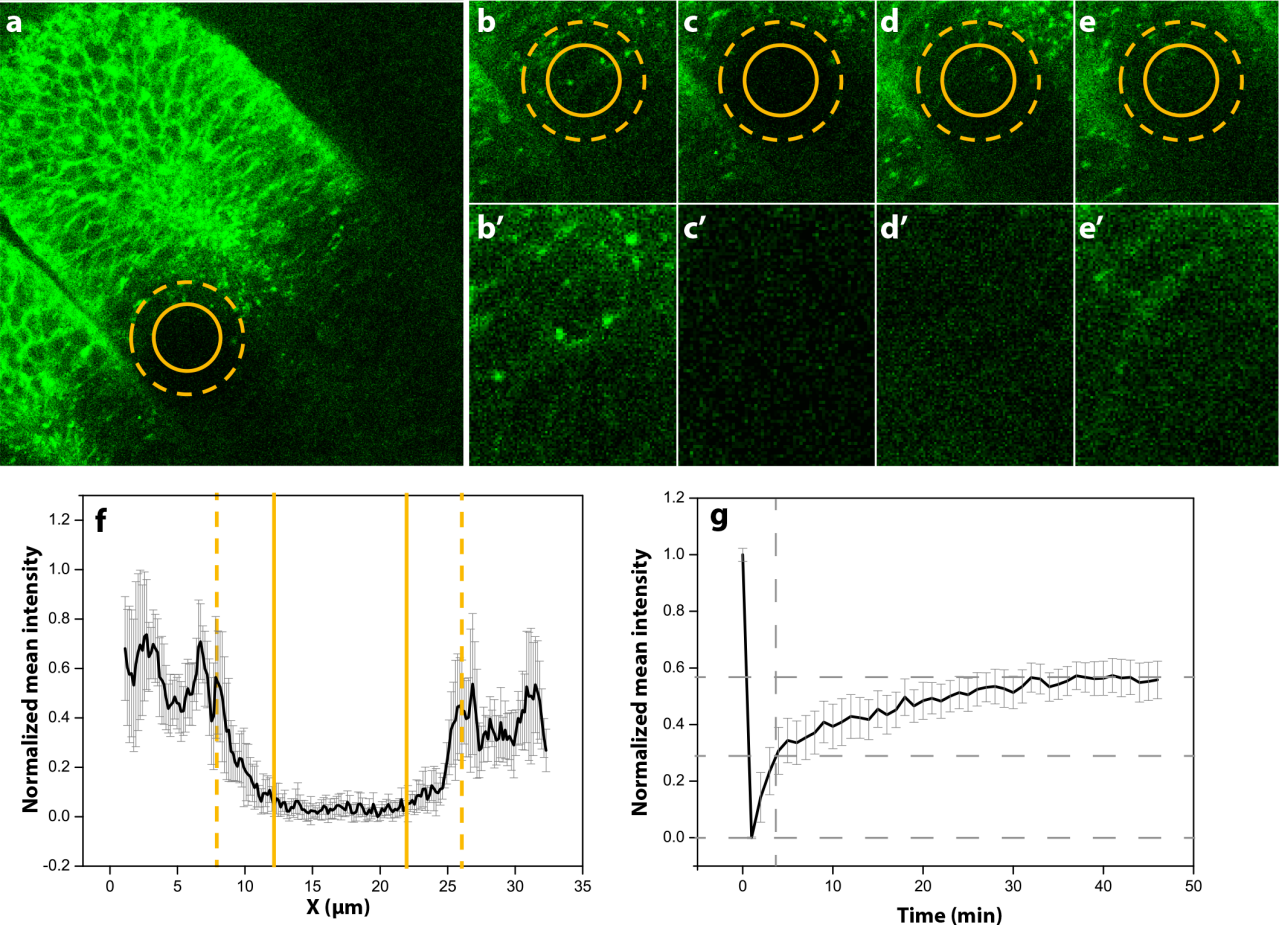
Determination of the Hh diffusion coefficient. (a) Part of the wing disc where the FRAP was made. The yellow solid circle shows the ROI selected to perform the photobleaching. The yellow dashed circle shows the photobleaching effective area. (b-d) Different time (T) points of the photobleached area. (b'-d') Detailed photobleached area where the differences in fluorescence can be observed. (b,b') Frame before the photobleaching. (c,c') Frame just after photobleaching. (d,d') T = 10 min. (e,e') T = 60 min. (f) Bleaching profile of the normalized mean intensity. Here can be observed the nominal diameter (2*r*_*n*_) between yellow solid lines (correspondent to the photobleached ROI) and the effective diameter (2*r*_*e*_), between yellow dashed lines, correspondent to the effective photobleached area. (g) FRAP recovery profile. The grey dashed lines show the half recovery time corresponding to one of the samples used in the experiment. The mean half recovery time is determined as τ_1/2_ = 7.12 min (*D*_*Hh*_ = 0.033 ± 0.006 μm^2^ s^-1^).

In order to determine the Hh effective diffusion coefficient, wing discs were dissected from larvae in which UAS-GFP:Hh [32] was driven in the *hh*-expression domain by a *hh*-GAL4 driver. In the FRAP experiment, the region of interest (ROI) (solid circle in Fig 3A) was photobleached and the recovery was observed (Fig 3B–3E). The bleaching does not only happen in the ROI, but also in the adjacent area (Fig 3F). From the FRAP profile we calculated the mean half recovery time τ_1/2_ = 7.12 min (Fig 3G). This corresponds to a mean diffusion coefficient of Hh of *D*_*Hh*_ = 0.033 ± 0.006 μm^2^ s^-1^ (see details in Materials). This value is similar to previous measurements for Wg in the wing disc [29], and Wg and Hh have previously been noticed to bear important similarities in their extracellular transport in the wing disc [33]. The characteristic length for Hh has been determined in the wing disc as 7 μm [34]. From the diffusion constant *D*_*Hh*_ = 0.033 μm^2^ s^-1^ that we determined, the Hh decay rate then follows as approximately δ_*Hh*_ = 6.7 × 10^−4^ s^-1^. We note that the Hh gradient has been shown to be dynamic during wing disc and ocellar complex development [35,36], something that we will ignore in this model as the effect can be expected to be minor for our model predictions, and it would require a major complication of the model as the Hh’s receptor Patched (Ptc) would need to be included explicitly.

### Detailed Eye Disc Model Reproduces Linear MF Movement and Growth Termination

**Fig 4:**
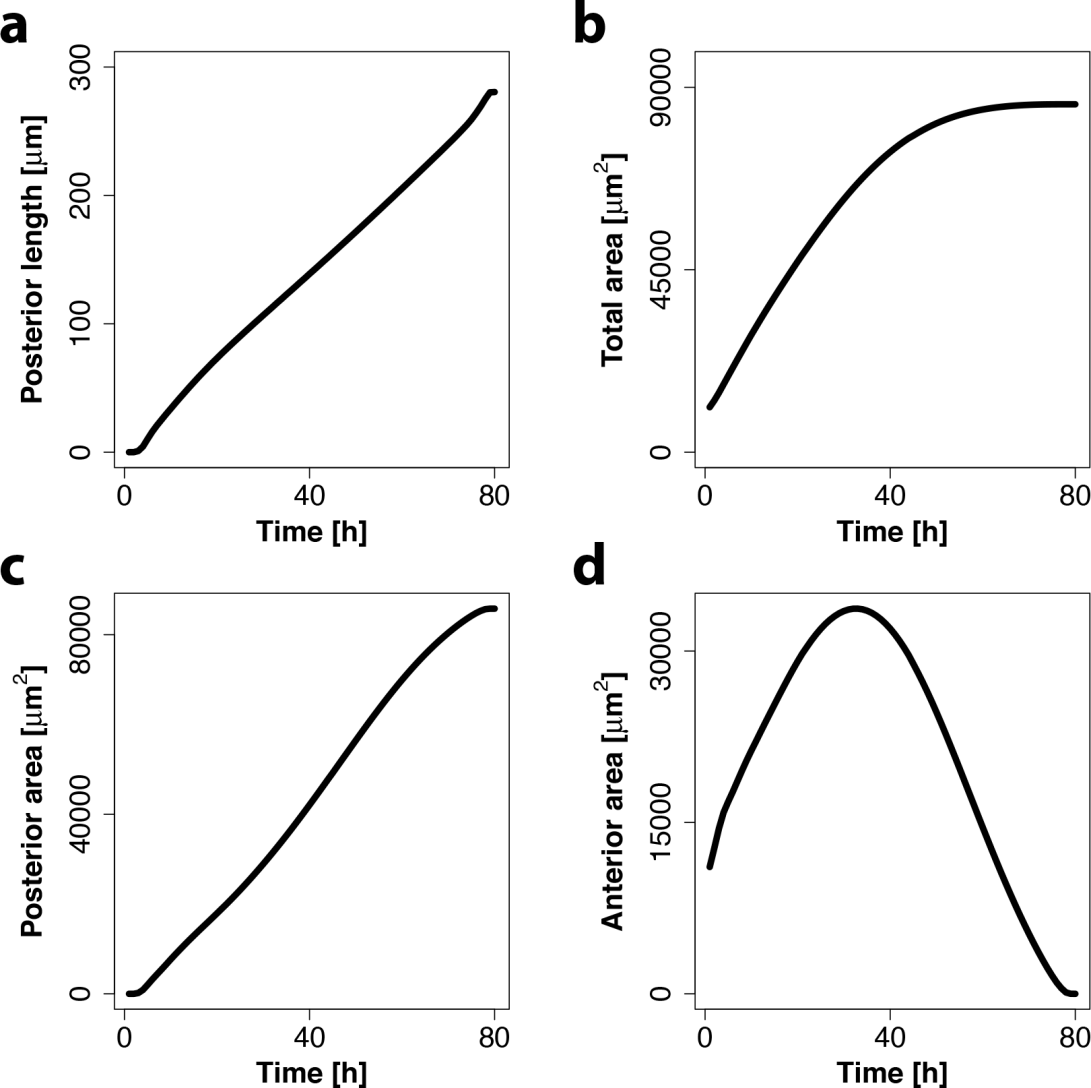
Eye disc model captures linear MF movement and growth termination. (a) Simulated progression of MF is approximately linear over time (black line). The speed of the MF movement is determined by a linear fit and is ≈3.4 μm h^-1^. (b-d) Simulated total area (b), posterior area (c) and anterior area (d) over time in the eye disc model show growth termination towards the end of development.

In agreement with previous measurements [9], the MF progresses linearly with time from the posterior towards the anterior side of the domain (Fig 4A). Moreover, the speed of MF progression agrees quantitatively. Here, we note that the experimental measurements report the MF position as the average posterior length at a given time point across the disc, while we monitor the MF position as the maximal posterior length at the dorso-ventral boundary to be able to use the previously determined eye disc growth rate in Eq 9 [9]. We have previously shown that the experimentally determined speed of 3.1 μm h^-1^ [9] then corresponds to 3.4 μm h^-1^ [24], as observed in our numerical simulations of the eye disc model.

The model also reproduces the observed growth dynamics (Fig 4B–4D). Thus, our simulations show an initial linear increase of the total area followed by a plateau phase (Fig 4B). During the plateau phase the anterior area is barely increasing and finally declines in parallel and at a similar rate as the posterior area is increasing due to the differentiation caused by the progression of the MF (Fig 4B–4D). As a result of this, the MF reaches at some point the anterior end of the eye disc which leads to growth termination due to the exhaustion of anterior progenitors.

**Fig 5:**
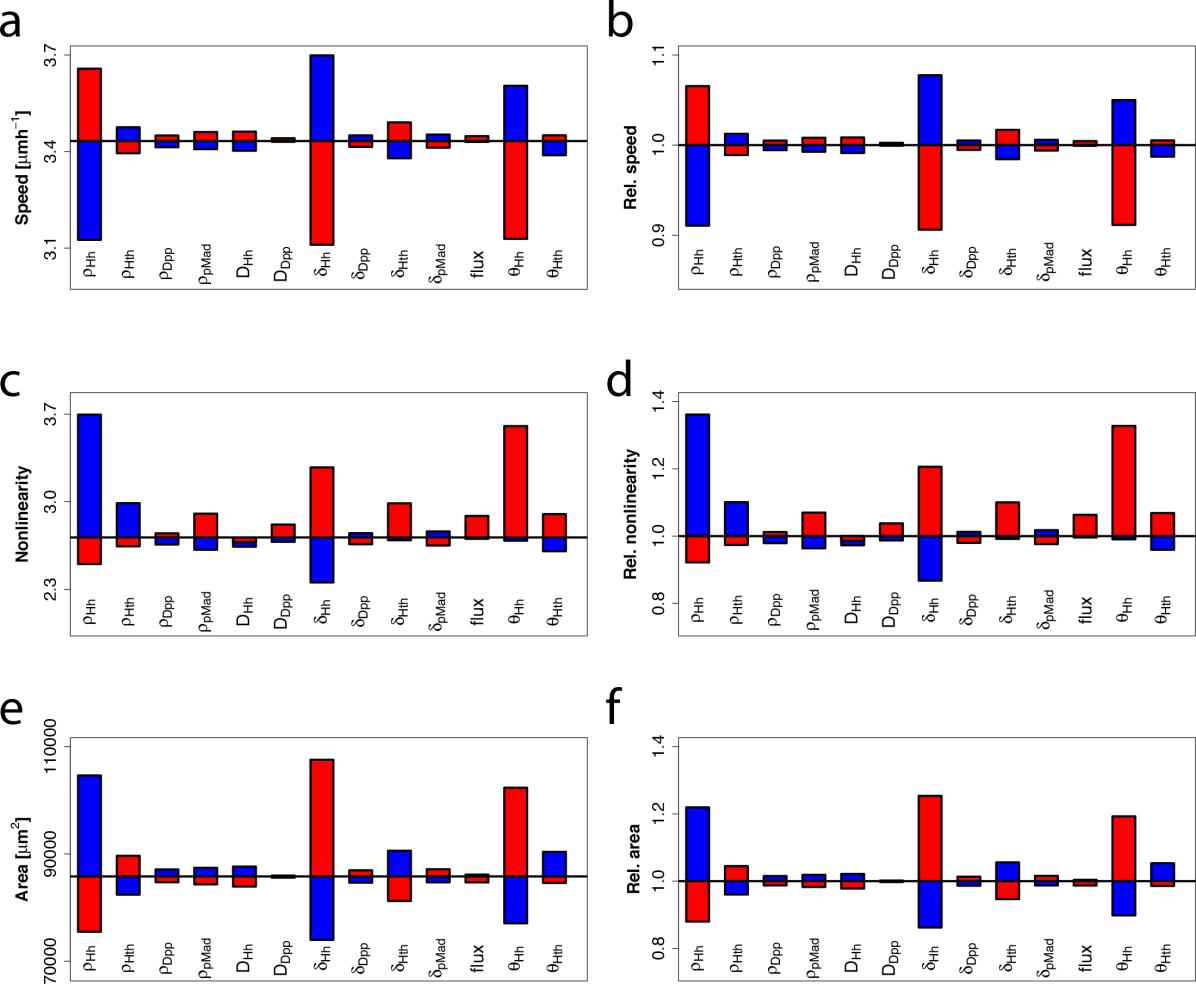
Effects of parameter changes on MF speed, nonlinearity and final eye disc area. Blue bars indicate a 1% decrease and red bars a 1% increase in parameter values compared with the wildtype (black line). In panels a, c, and e, the absolute values for speed, nonlinearity and area of the resulting eye discs are shown whereas in b, d, and f, the same data is shown in relation to the wildtype values.

In our numerical simulations, the nonlinearity and speed of the MF movement (measured by the root-mean square error and the slope of a linear fit of the posterior length over time) as well as the final eye disc size heavily depend on the choice of parameters. In order to quantify the effect of parameter changes in the model we increased (Fig 5, red boxes) or decreased (Fig 5, blue boxes) single parameters by 1%. An increased impact of Hh, either achieved by a higher production rate *p*_*Hh*_, a lower degradation rate *δ*_*Hh*_ or a lower concentration threshold for differentiation *θ*_*Hh*_, clearly has the strongest effect in speeding up the MF (Fig 5A and 5B). At the same time, an increased impact of Hh also increased the linearity of the MF movement (Fig 5C and 5D). As a result of the increased MF speed, the anterior tissue has less time to proliferate and therefore the final eye disc area is smaller (Fig 5E and 5F). In contrast to this, an increased impact of Hth (high production rate *p*_*Hth*_, low degradation rate *δ*_*Hth*_ or lower concentration threshold for differentiation *θ*_*Hth*_) decreases the MF speed and increases its linearity (Fig 5A–5D). As a result of the slower MF movement the anterior area has more time to proliferate and therefore the final total area increases (Fig 5E and 5F). An increased impact of both Dpp and pMad has generally a smaller effect but nevertheless leads to an increase in the MF speed and in the nonlinearity of the MF movement and to a decrease in the final area (Fig 5).

### Growth and MF Dynamics in Several Mutants Can Be Explained by the Eye Disc Model

**Fig 6:**
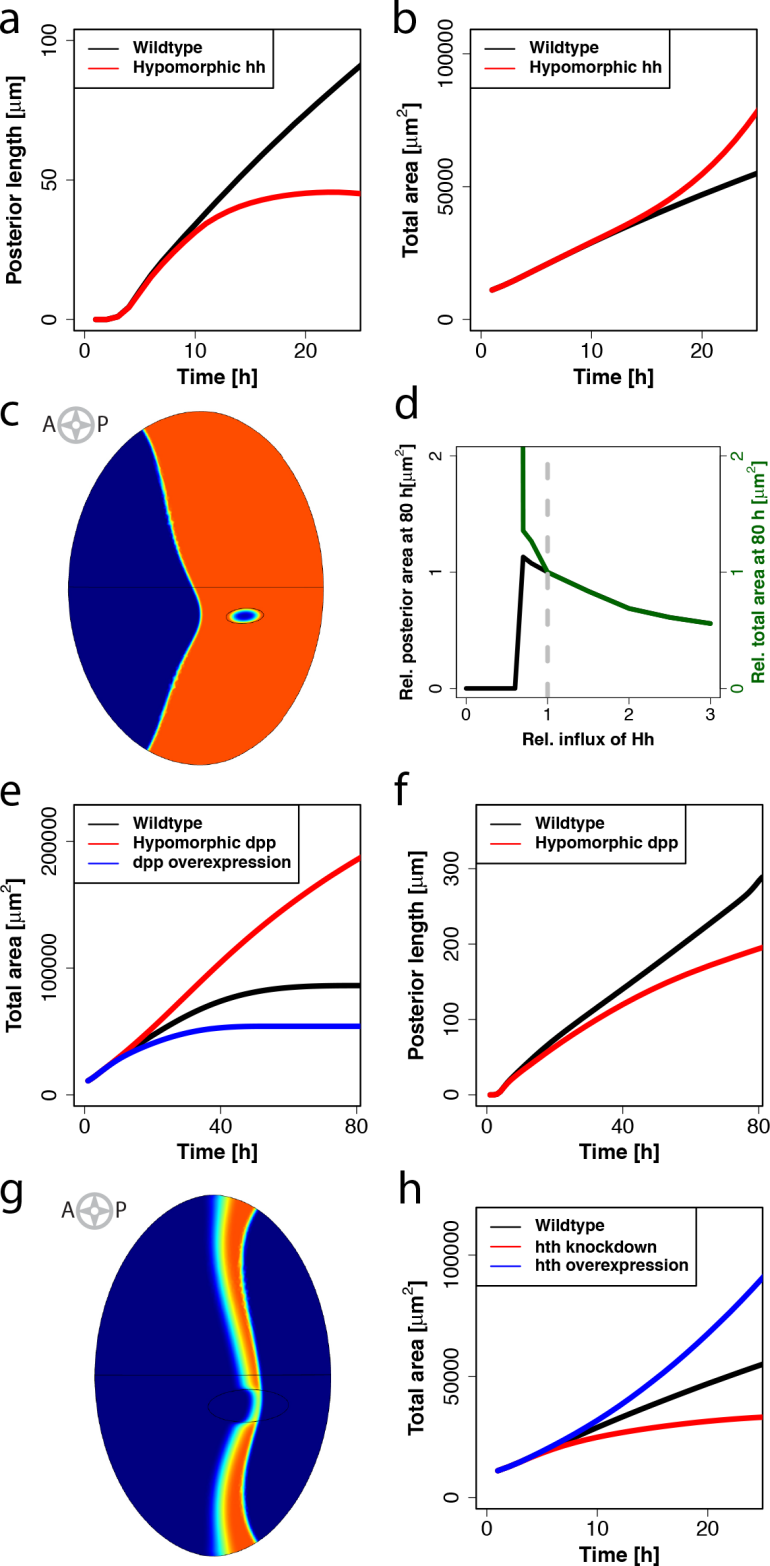
Eye disc model can reproduce several mutant phenotypes. (a,b) Comparison of the MF movement (a) and the total area over time (b) between a simulation using the original parametrization (indicated by the black line) and a simulation using a reduced hh production rate (indicated by the red line). In the case of the simulation representing the hypomorphic hh mutant the MF is clearly slower and eventually stops (a), while the total area is overgrowing (b). (c) Simulation of a clone where the hh production rate is set to zero at t = 55h. Towards the center of the clone the tissue is not differentiated (blue color) despite residing within the posterior area (colored in orange). (d) Posterior (indicated by the black line) and total (indicated by the green line) eye disc sizes in relation to the wildtype (indicated by the dashed grey line) at different relative influxes of Hh from the posterior margin. (e,f) Comparison of the total area over time (e) and the movement of the MF (f) between a simulation using the original parametrization (black line) and a simulation using a reduced (red line) dpp production rate. In panel e an additional simulation with increased dpp production rate (blue line) is compared. (g) Simulation of a clone where the hth production rate is increased at t = 20h. The MF (indicated by orange color) shows a retardation in the clone. (h) Total area over time in a numerical simulation representing the wildtype phenotype (black line) and a simulation using a reduced (red line) or increased (blue line) hth production rate.

Experiments show that the speed of the MF and/or the size of the adult eye are severely affected in Hh, Dpp and Hth mutants. We were therefore interested to see to what extent our eye disc model is able to explain these observed effects. Mutations that reduce Hh activity in the eye disc result in a severe slowdown or stop of MF progression and eye size reduction [17,37–39]. In our simulations, reduction of the Hh production rate indeed results in a decreasing speed and an eventual stop of MF movement (Fig 6A). Furthermore we observe that the predicted total area of the eye disc overgrows (Fig 6B), because the differentiation rate of the proliferating area caused by the furrow is much smaller. This excess of undifferentiated progenitors do not make into the adult head, as *hh* mutants show smaller eyes but no abnormal overgrowths. This suggests that there must be some additional control of the anterior area that is not included in the eye disc model, e.g. downregulation of the growth rate or increased cell death in the absence of Hh signaling. The latter is supported by the observation that there is abundant cell death in *hh*-mutant discs anterior to the MF [16,17]. Furthermore, it was shown that large *hh* signaling-mutant clones in the eye disc showed a disrupted organization of photoreceptors towards the center of the clone [17]. Interestingly, we can see that also in simulations of these clones the Hh concentration in the center is not sufficient to differentiate these cells (Fig 6C). It is well known that Hh is required for the initiation of the MF, and removal of *hh* expression in the margin thus prevents MF initiation and eye formation [40,41]. Complete removal of Hh signaling in the simulations precludes production of Dpp and since *hth* can no longer be downregulated, no MF starts (Fig 6D, zero influx). On the other hand, a small reduction in the Hh influx compared to wild type increases eye size because cells have more time to proliferate before the MF is initiated (Fig 6D).

Similar to the phenotype of discs with hypomorphic *hh* alleles, eye discs harboring a hypomorphic *dpp* allele have very small adult eyes (*dpp* ^*blk*^: 100–200 ommatidia develop instead of 700–800 in the wild type) [37]. In contrast to this, our simulations predict an overgrowth of the total area (Fig 6E, red line). Again this is because in our simulations the MF moves more slowly (Fig 6F), thus leaving more time to anterior cells to proliferate, which finally results in larger eyes. The predicted effect of *dpp* on furrow movement is supported by experiments that show that the MF slows down in clones mutant for the *dpp* signal transduction pathway [42–44]. The difference in the final size of the eye disc between model and experimental data can be explained by the fact that we did not include any effect of *dpp* on cell survival in the model and therefore it is not able to capture the experimentally observed excessive cell death in the ventral regions of the mutant eye discs [37].

Experiments show that *hth*-overexpressing clones slow down MF movement, something that we can also observe in our numerical simulations (Fig 6G). It has also been shown that downregulation of Hth levels mediated through the expression of an RNAi construct in the eye primordium (using an *eyeless* driver) leads to a reduction in the adult eye size [20]. In our simulations, a decrease of the Hth production rate indeed leads to smaller eyes (Fig 6H, red line). However, we observe overgrowth in the total eye area if we increase the *hth* production rate, because proliferating cells are delayed in differentiating (Fig 6H, blue line). Eye overgrowth in response to hth overexpression has not been experimentally observed and therefore this result might suggest that a fundamental link between patterning and growth, such as increased cell death in the progenitor population in mutant eye discs, is not included in the model. Furthermore, it is known that excessive Hth, which is not bound to the protein Extradenticle (Exd), is degraded [45]. Therefore, contrary to the assumption of the model, the overexpression of Hth might not have an impact on the total Hth concentration if the steady state Hth concentration is close to its maximum.

### The Simulated Dpp Gradient Is Not Scaling in the Anterior Compartment

**Fig 7:**
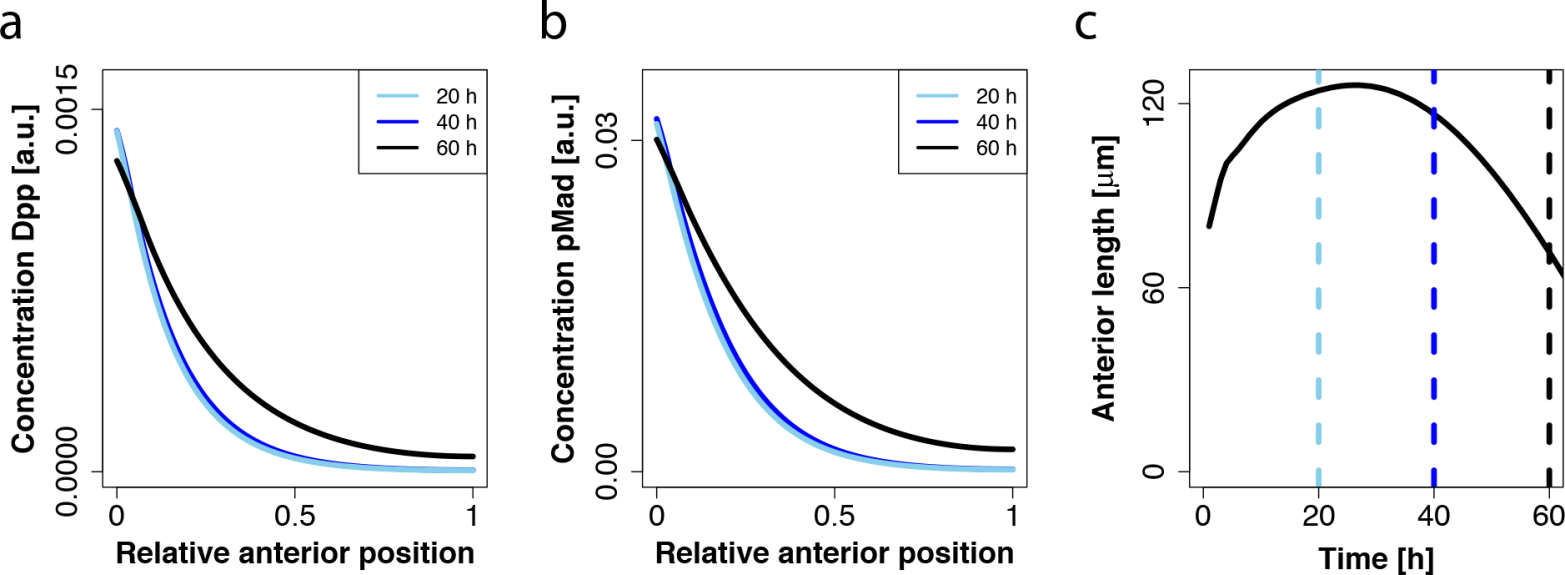
The simulated Dpp gradient is not scaling in the anterior compartment. (a,b) The simulated spatial profiles of Dpp (a) and pMad (b) in a developing eye disc at 20h (light blue), 40h (dark blue) and 60h (black). (c) Anterior length over time (black solid line). Vertical dashed lines indicate time points corresponding to (a,b).

Recently, it was reported that the gradients of the Dpp targets pMad and *hairy* scale with the length of the anterior domain [9]. As the anterior length is initially increasing and then decreasing, the Dpp gradient would have to initially expand and later retract. In order to compare this observation with the results of our model we normalized the simulated Dpp and pMad gradients from three time points with respect to the anterior domain length. Indeed the relative profiles at 20 hours and 40 hours overlapped almost perfectly, while the relative profile at 60 hours was expanded (Fig 7A–7C). However, in our model, the overlap is not a result of an adaptation of the gradient to the anterior length, but the result of a constant decay length of the gradient and very similar anterior domain length at 20 and 40 hours, such that a constant gradient also overlaps after normalization (Fig 7C). At 60 hours the anterior length is much shorter and the gradient therefore appears more expanded (Fig 7C). This cannot be explained by a change in the amplitude, since for both Dpp as well as pMad the maximal concentration is relatively stable over time in the simulations (Fig 7A and 7B). We conclude that our model cannot explain the observed scaling of the pMad and *hairy* gradients.

## Discussion

How growth and differentiation are integrated to ensure the proper development of tissues is still an open question. Here we developed a model for the *Drosophila* eye imaginal disc on a 2D growing domain that is based on a simple reaction network. We measured the two key unknown parameters, the Hh diffusion coefficient and the Hth degradation rate. We then investigated the behavior of our model and found that it can reproduce the initiation and linear progression of the MF. The linearity and speed of the MF movement are an important property for growth control because they determine the time for which progenitor cells on the anterior domain of the eye disc can proliferate. Interestingly, our model can qualitatively explain mutant phenotypes that are linked to changes in MF speed, which suggests that a very simple reaction network is sufficient to control and orchestrate the movement of the morphogenetic furrow. The reason for this is that the system is irreversibly bistable and that Hh and Dpp can diffuse. Both properties taken together generate a travelling wave, which in this case can be observed as the MF.

However, we can also see that our model cannot explain some of the mutant phenotypes linked to the final eye size, which suggests that the differentiation wave caused by the movement of the MF is insufficient to explain growth control in the eye disc and that there must be additional layers of regulation between patterning and growth. One such layer of control might be the compensatory increase in cell death in response to a lack of differentiation or proper MF progression. It was shown that *dpp*^*d-blk*^ discs, in which *dpp* production at the MF is lacking and the MF halts, show an increment of cell death in the ventral half of the eye disc [37]. The same effect is not limited to *dpp*, but can also be observed in hth-overexpressing discs and *hh* mutant discs. However, this effect seems to be limited to situations where the whole disc is mutant and is not observed in mutant clones, suggesting that Hh and Dpp might act as survival factors and are crucial in determining the probability of cell death in the progenitor cells. In fact, it has been described that Dpp is used as a survival signal in the wing disc [46]. The Dpp gradient has been suggested as an additional level of control. In particular, it has been proposed that the relative temporal change in the concentration levels of Dpp has a direct influence on growth and ensures non-uniform growth and growth termination in the eye disc [9], and uniform growth and growth termination in the wing disc [9,37,47]. However, the relevance of Dpp on *Drosophila* wing disc growth is currently being debated. Dpp has been reported to have no major impact on growth in the lateral regions of the *Drosophila* wing disc [48,49], and recent results even suggest that Dpp has a minor impact on wing disc growth during third instar [50].

Finally, we investigated the scaling of the Dpp gradient in the anterior side. In contrast to experimental reports [9], we do not observe scaling in our model. Scaling in the eye disc is particularly interesting because at later stages the anterior length (i.e. the width of remaining progenitor area) is decreasing and the gradient would therefore have to shrink in order to scale. Such shrinkage cannot be explained by previous scaling models from the wing imaginal disc [4,30,51]. A novel scaling mechanism would thus need to be in place that ensures scaling of the Dpp gradient in the eye disc. To define such scaling mechanism, it will be important to also obtain quantitative measurements of the Dpp gradient itself, in particular in late stages of eye disc development when the Dpp gradient is supposed to shorten.

Our model for the Drosophila eye disc is a first step on the way to mechanistically understand the interplay between patterning and growth during development. Our model explains many key observations but also highlights important gaps in our understanding. In particular, our results show that our model is currently lacking important regulatory cues on both cell survival and cell proliferation. Additionally, the model mainly focuses on the major players along the anterior-posterior axis and neglects some factors acting along the DV axis, such as the prominent antagonist of eye differentiation, the Drosophila WNT-1 homologue Wingless (Wg). Wg is produced from the anterior dorsal and ventral margins of the disc to restrict MF initiation at the posterior margin [38]. It is known that ectopic *hth* can repress cell differentiation independently of Wg [23], suggesting that Hth might work downstream of Wg. Including Wg explicitly in future versions of the model might lead, for example, to a better understanding of the hypomorphic *dpp* mutant that shows abnormal cell death on the ventral side. At the same time, more experimental work has to go into dissecting the complex network that seems to have an influence in controlling growth.

## Methods

### *D*. *melanogaster* Strains and Genetic Manipulations

*Drosophila* cultures were raised at 25°C. The strain *w*^*1118*^ (Flybase identifier: FBal0018186) was used to determine the quantitative profiles of Hth, Eya and pMad. *YFP*:*hth* (CPTI-000378) is a protein trap from the Cambridge protein trap project, FlyProt [52]. UAS-GFP:Hh was driven by a *hh-GAL4* driver as in [53].

### Immunofluorescence and Imaging

Eye imaginal discs were dissected and fixed according to standard protocols, except that primary antibodies were incubated in a hypotonic buffer solution (0.75xPBS with 0.1% TritonX-100) to allow a better separation of nuclei and their segmentation. Primary antibodies used were rabbit anti-pMad, 1/5000 ([54]; kindly provided by G. Morata, CBMSO); guinea pig anti-Hth (GP52), 1/50 ([55]); mouse anti-Eya (eya10H6), 1/100 (Developmental Studies Hybridoma Bank (Iowa University). Fluorescently labeled secondary antibodies were from Molecular Probes. Discs were counterstained with DAPI to mark nuclei. Images were obtained on an SPE Leica confocal setup and nuclei were subsequently segmented; as disc epithelia present folds, eye discs images were computationally stretched to obtain a correct measure of distances.

### Image Segmentation and Correction for Disc Folding

Stacks of regularly spaced images were acquired by laser confocal microscopy (LCM) such that, on average, each nucleus was contained in two or three contiguous confocal planes. To this end, a narrow pinhole and a z-step of 1.521 μm were used for DAPI signal acquisition. The other channels were acquired subsequently with a pinhole that spanned exactly 1.521 μm in order to avoid over- and under-sampling upon quantification (objective ACS-APO 40x, numeric aperture 1.15).

We developed a new software tool, iFLIC, to perform the segmentation and stretching of folded discs. A detailed description of the software will be published elsewhere. In brief, the software first segmented the nuclei in 3D by identifying all pixels that belonged to a given nucleus within the densely clustered images. Segmentation was based on bandpass filtering that made use of otsu thresholds. Subsequently, the point closest to the center of each nucleus was determined based on the intersection of solid spheres that were fitted into the segmented nuclei. Once the centers of the nuclei had been estimated, ellipsoids were fitted by altering both the lengths of the semi-axes and their orientation until the ellipsoid that covered the segmented pixels with the maximum volume had been found. Boundaries were expanded by flooding with equal chances for all ellipsoids, up to the point that no white voxels were left (complete assignation). Signals were quantified inside the nuclei as the mean pixel intensity (in 8-bit scale) for each channel.

Eye imaginal discs are frequently folded, which affects the reconstruction of reliable gene expression profiles. To correct for the deviations that are introduced by the pleating we developed an algorithm to computationally stretch out the tissue, using the centroid coordinates obtained from the previous segmentation. The epithelium is pseudostratified, and the nuclei are therefore positioned at random along the apical-basal axis. To obtain a distance measurement, we mapped the positions of the nuclei to the apical surface. In a first step the z-coordinate from an orthogonal user-selected system was estimated by a gaussian kernel whose bandwidth had been previously determined by generalized cross validation (GCV) [56]. The gaussian kernel mapped the z-coordinate at regular intervals in the current z-projection in order to check the z-coordinate, thus providing an continuous and differentiable surface that passes through nuclei estimated centroids. Once the surface had been determined within the 3D dataset, we determined every nucleus footpoint that maps onto the surface. With this information at hand, we could then calculate the real surface distances of the cells, to which each nucleus belongs.

Finally, the immunofluorescence intensity profiles were obtained from narrow stripes of stretched sets of nuclei oriented along the anterior-posterior axis.

### Fluorescence Recovery After Photobleaching (FRAP)

Two different experiments were performed using Fluorescence Recovery After Photobleaching (FRAP). The first one is a bleach-chase experiment [31,57] performed to calculate the degradation rate of Hth. The remaining was performed to determine the diffusion coefficient of the morphogen Hh.

The experimental preparation was equally done for both experiments. Imaginal discs were dissected in SF-900 medium at room temperature, and transferred to a medium-containing well with a glass coverslip bottom. The samples were maintained at room temperature, which is fixed at 21°C.

The data analysis was done using different software applications. For the imaging analysis ImageJ v.1.47f was used; the statistics was done using the Microcal Origin v.8.1 software.

### Experimental Determination of the Degradation Rate of Hth

For these experiments, late L3 eye-antennal discs of a YFP:Hth protein trap strain (CPTI-000378, Cambridge protein trap project, FlyProt [52]) were used. In the FRAP experiment for determining the Hth degradation rate, the regions of interest (ROIs) (Fig 2A and 2B in yellow rectangles as examples) were photobleached using a 30s pulse of 488nm Argon laser, with 100% laser power and 100% transmission during. The recovery was observed by exciting YFP in the sample with a 488nm Argon laser with laser power 20% and transmission 14–25%. This laser is installed on a confocal microscope Leica SP5 with 63x(1.40) HCX PL APO CS objective using Leica OIL 11-513-859, zoom 1.4–1.7X, gain ~1200. The movies had a duration of 120 min, with one frame captured every 5 minutes. Each frame was the average of three scans. In order to control cell movement along the z axis, a z-stack 6.3 μm wide (approximately a cell diameter) was captured divided into 3 steps separated by 2.1 μm. In this way, it was possible to capture small cell movements. The number of samples taken to perform this analysis was N = 6.

As the images are noisy it is quite difficult to perform a clear nuclei tracking. Therefore, the measures were done by acquiring mean intensities from small areas of 4–5 nuclei extension. Before performing the bleach-chase analysis, we averaged the z-projection. The projection step smoothens out noise and maps small nuclear movements into one frame.

The bleach-chase protocol, as previously described [31,57], is based on a comparison of the intensity in the bleached region (yellow rectangle in Fig 2A) and the unbleached region (red rectangle in Fig 2A) over time. In the following we give a brief summary of the procedure. As a result of bleaching, part of the protein concentration becomes invisible. The total protein concentration, *P*_*T*_, is the sum of the visible protein *P*_*v*_ and the invisible protein *P*

$$P_{T}(t)=P_{v}(t)+P(t)$$ (10)

The invisible protein is only produced during the bleaching process; both proteins degrade according to the following equation:

$$\frac{dP(t)}{dt}=-\delta_{Hth}\cdot P(t)$$ (11)

As *P* is invisible it cannot be measured directly but the degradation rate coefficient of the visible and invisible protein is the same so, it can be determined by comparing the invisible and the visible intensity evolution.

$$\frac{dP(t)}{dt}-\frac{dP_{v}(t)}{dt}=-\delta_{Hth}\cdot (P(t)-P_{v}(t))$$ (12)

The solution to this equation is:

$$P(t)-P_{v}(t)=(P(0)-P_{v}(0))\cdot exp(-\delta_{Hth}\cdot t)$$ (13)

with *P(0)* and *P*_*v*_*(0)* being the intensity values for visible and invisible proteins at time *t* = 0. Taking the logarithm we further obtain

$$\text{ln}(P(t)-P_{v}(t))=\text{ln}(P(0)-P_{v}(0))-\delta_{Hth}\cdot t$$ (14)

The degradation rate coefficient can thus be obtained by plotting the logarithm of the difference in intensity of unbleached and bleached regions versus time, and linearly. The slope of the fitted line gives −*δ*_*Hth*_. The protein half-life can then be obtained as

$$\tau_{1/2}=\frac{\ln2}{\delta_{Hth}}$$ (15)

The time series for the bleach-chase analysis of Hth is shown in Fig 2B. The slope of the fitted line yields the degradation rate, *δ*_*Hth*_ = (6.97 ± 5.00) 10^−5^ s^-1^, which corresponds to a Hth protein half-life of τ_1/2_ = 2.77 h.

### Experimental Determination of the Hh Diffusion Coefficient

In this experiments, wing discs were dissected from larvae in which UAS-GFP:Hh [32] was driven in the *hh*-expression domain by a *hh*-GAL4 driver. Wing discs were used due to the larger domain of Hh expression and the straight border between expressing and non-expressing cells. In the FRAP experiment for determining the Hh diffusion coefficient, the ROI (solid circle with a radius of 5 μm in Fig 3A) was photobleached for 40s using an Argon laser 488nm with laser power 100% and transmission 100%. The recovery was observed by exciting GFP in the sample with an Argon laser 488nm with laser power 20% and transmission 14–25%, pinhole 0.99. The laser was installed on a confocal microscope Leica SP5 with 63x(1.40) HCX PL APO CS objective using Leica OIL 11-513-859, zoom 3.0–4.2x, gain ~1200. The movies recorded 60 minutes of intensity recovery, taking one frame every 2 minutes. Each frame was the average of three scans. In order to control for cell movement along the z axis, a z-stack 6.3 μm wide (approximately one cell diameter) was captured, divided into 3 steps separated 2.1 μm. In this way, it was possible to capture small cell movements. The number of samples taken to perform this analysis was N = 6.

The image analysis was performed following Kang et al. simplified equation to obtain diffusion coefficients from confocal FRAP data [58]. Here the diffusion coefficient is defined as:

$$D=\frac{r_{e}^{2}+r_{n}^{2}}{8\cdot \tau_{1/2}}$$ (16)

where *r*_*n*_ is the nominal radius (ROI radius), *r*_*e*_ is the effective radius (spreading radius of postbleached profile) and *τ*_1/2_ is the half time of the recovery. In order to calculate *r*_*e*_, the bleaching profile (Fig 3F) can be approximate by a Gaussian profile fitting it to the following expression:

$$f(x)=1-K\cdot exp\left(\frac{-x^{2}}{r_{e}^{2}}\right)$$ (17)

*K* and *r*_*e*_ can be obtained using a nonlinear least-squares fitting routing (nlinfit.m) available in MATLAB.

These parameters can also be obtained by applying a direct protocol. First, *K* can be determined from the bleaching depth in the normalized postbleach profile as referred to in Fig 3B. Then, the half width of cross-sections between the horizontal line at the height of 0.86K from the bottom of the postbleach profile (Fig 3B) and the postbleach profiles yields *r*_*e*_ without involving any fitting (Fig 3B).

To measure *τ*_1/2_ from the FRAP data a linear interpolation method was used. The FRAP data can be defined as a time dependent function *F*: {*F*(0),*F*(*t*_1_),*F*(*t*_2_),…,*F*(*t*_*n*_)} such that *F*(0) = *F*0 and *F*(*t*_*n*_) = *F*^∞^. The fluorescence intensity at half of recovery is defined as *F*_1/2_ = (*F*0 + *F*^∞^)/2. If *F*(*t*_*k*_) = *F*_1/2_ for some *t*_*k*_ then the half-recovery time follows as *τ*_1/2_ = *t*_*k*_. If *F*(*t*_*k*_) < *F*_1/2_ < *F*(*t*_*k*+1_) it is defined as:

$$\tau_{1/2}=t_{k}+\frac{F_{1/2}-F(t_{k})}{F(t_{k+1})-F(t_{k})}(t_{k+1}-t_{k})$$ (18)

The result of this calculation is shown for one of the samples that were used in the experiment (Fig 3G). The calculated mean half recovery time is τ_1/2_ = 7.12 min. After applying equation Eq.16 to every sample, the mean diffusion coefficient of Hh is obtained as *D*_*Hh*_ = 0.033 ± 0.006 μm^2^ s^-1^.

### Boundary Condition for Hh

As previously stated in Eq 6 the boundary condition for Hh depends on a spatial function Λ *Λ*(*x*) and a temporal function *τ*(*t*). We know that initially Hh influx only occurs at the posterior margin. During eye disc growth also the part of the boundary with a non-zero Hh influx is growing, and therefore *Λ*(*x*) is defined on a relative domain. If we set the x-axis to zero at the posterior end of the eye disc, pointing towards the anterior region, then the anterior-most point equals the total AP length *L*_*AP*_(*t*). We then define our spatial function as 1 for the part of the boundary where the x-position is within the first 20% of the distance between 0 and the total AP length:

$$\Lambda (x)=\left\{ \begin{array}{c} \begin{array}{ccc} 1 & \text{if} & x\leq 0.2\cdot L_{AP}(t) \end{array}\\ \begin{array}{ccc} 0 & \text{if} & x>0.2\cdot L_{AP}(t) \end{array} \end{array}\right.$$ (19)

This is also illustrated in Fig 1A (orange highlighted margin). The flux of Hh into the domain will decrease over time as the eye disc becomes filled with Hh (also as a result of Hh production by the differentiated cells) such that the Hh gradient between the margin and the eye disc vanishes. We could have incorporated this in the simulation by introducing a finite size margin. However, for numerical efficiency we choose a time-dependent function that qualitatively reproduces the decreasing influx of Hh:

$$\tau (t)=\left\{ \begin{array}{c} \begin{array}{ccc} 1 & if & t<10h \end{array}\\ \begin{array}{ccc} 1-\frac{t-10h}{1h} & if & 10h\leq t\leq 10.5h \end{array}\\ \begin{array}{ccc} 0 & if & t>10.5h \end{array} \end{array}\right.$$ (20)

### Software for Numerical Simulations

The equations were solved with finite element methods (FEM) as implemented in COMSOL Multiphysics 4.3b. In COMSOL both the Creeping Flow as well as the Moving Mesh module, which is an implementation of the ALE method, were activated. For all simulations the PARDISO solver with multithreaded nested dissection as the preordering algorithm and an automatic scheduling method was used. Pivoting perturbation was set to 1E-8. In the time stepping menu, the BDF method with a maximum step size of 250 seconds and an event tolerance of 0.01 was chosen. The initial mesh was generated with a maximum element size of 5E-6 m and a minimum element size of 2E-8 m. The maximum element growth rate was set to 1.1 and the curvature factor to 0.2. Remeshing was automatically enforced when the minimum mesh element quality was below the threshold of 0.3. Other options were not changed from standard settings.

## Supporting Information

S1 Fig Measured and simulated concentration profiles. (a-c) Comparison of measured pMad (a), Hth (b) and Eya (c) profiles to the simulated gradients at 25h. Black lines indicate the estimated mean concentration over 60 bins created from the experimental data, dark grey areas indicate the estimated standard error and light grey areas indicate the 90% confidence interval for pMad, Hth, and Eya.
(TIF)

S1 Table Parameter values. The table summarizes all parameter values used in the model along with the evidence. All components *i* have the same production rate *p*_*i*_.
(DOCX)

## References

[1] Kim J, Sebring A, Esch JJ, Kraus ME, Vorwerk K, Magee J, et al Integration of positional signals and regulation of wing formation and identity by Drosophila vestigial gene. Nature. 1996;382: 133–8. 10.1038/382133a0 8700202

[2] Zhang Y, Kalderon D. Regulation of cell proliferation and patterning in Drosophila oogenesis by Hedgehog signaling. Development. 2000;127: 2165–76. Available: http://www.ncbi.nlm.nih.gov/pubmed/10769240 10769240 10.1242/dev.127.10.2165

[3] Baker NE. Patterning signals and proliferation in Drosophila imaginal discs. Curr Opin Genet Dev. 2007;17: 287–93. 10.1016/j.gde.2007.05.005 17624759

[4] Wartlick O, Mumcu P, Kicheva a, Bittig T, Seum C, Jülicher F, et al Dynamics of Dpp signaling and proliferation control. Science. 2011;331: 1154–1159. 10.1126/science.1211373 21385708

[5] Heberlein U, Singh CM, Luk a Y, Donohoe TJ. Growth and differentiation in the Drosophila eye coordinated by hedgehog. Nature. 1995;373: 709–711. 10.1038/373709a0 7854455

[6] Amore G, Casares F. Size matters: The contribution of cell proliferation to the progression of the specification Drosophila eye gene regulatory network. Dev Biol. 2010;344: 569–577. 10.1016/j.ydbio.2010.06.015 20599903

[7] Bessa J, Gebelein B, Pichaud F, Casares F, Mann RS. Combinatorial control of Drosophila eye development by eyeless, homothorax, and teashirt. Genes Dev. Cold Spring Harbor Laboratory Press; 2002;16: 2415–2427. 10.1101/gad.1009002 12231630 PMC187435

[8] Peng HW, Slattery M, Mann RS. Transcription factor choice in the Hippo signaling pathway: homothorax and yorkie regulation of the microRNA bantam in the progenitor domain of the Drosophila eye imaginal disc. Genes Dev. 2009;23: 2307–19. 10.1101/gad.1820009 19762509 PMC2758742

[9] Wartlick O, Jülicher F, Gonzalez-Gaitan M. Growth control by a moving morphogen gradient during Drosophila eye development. Development. 2014;141: 1884–93. 10.1242/dev.105650 24757005

[10] Penton A, Selleck SB, Hoffmann FM. Regulation of cell cycle synchronization by decapentaplegic during Drosophila eye development. Science. 1997;275: 203–6. Available: http://www.ncbi.nlm.nih.gov/pubmed/8985012 8985012 10.1126/science.275.5297.203

[11] Horsfield J, Penton A, Secombe J, Hoffman FM, Richardson H. decapentaplegic is required for arrest in G1 phase during Drosophila eye development. Development. 1998;125: 5069–78. Available: http://www.ncbi.nlm.nih.gov/pubmed/9811590 9811590 10.1242/dev.125.24.5069

[12] Firth LC, Bhattacharya A, Baker NE. Cell cycle arrest by a gradient of Dpp signaling during Drosophila eye development. BMC Dev Biol. 2010;10: 28 10.1186/1471-213X-10-28 20214806 PMC2846880

[13] Bittig T, Wartlick O, Kicheva A, González-Gaitárr M, Jülicher F. Dynamics of anisotropic tissue growth. New J Phys. 2008;10: 63001 10.1088/1367-2630/10/6/063001

[14] Wiersdorff V, Lecuit T, Cohen SM, Mlodzik M. Mad acts downstream of Dpp receptors, revealing a differential requirement for dpp signaling in initiation and propagation of morphogenesis in the Drosophila eye. Development. 1996;122: 2153–2162. Available: http://www.ncbi.nlm.nih.gov/pubmed/8681796 8681796 10.1242/dev.122.7.2153

[15] Bonini NM, Leiserson WM, Benzer S. The eyes absent gene: genetic control of cell survival and differentiation in the developing Drosophila eye. Cell. 1993;72: 379–95. Available: http://www.ncbi.nlm.nih.gov/pubmed/8431945 8431945 10.1016/0092-8674(93)90115-7

[16] Ma C, Zhou Y, Beachy P a, Moses K. The segment polarity gene hedgehog is required for progression of the morphogenetic furrow in the developing Drosophila eye. Cell. 1993;75: 927–938. 10.1016/0092-8674(93)90536-Y 8252628

[17] Heberlein U, Wolff T, Rubin GM. The TGF?? homolog dpp and the segment polarity gene hedgehog are required for propagation of a morphogenetic wave in the Drosophila retina. Cell. 1993;75: 913–926. 10.1016/0092-8674(93)90535-X 8252627

[18] Curtiss J, Mlodzik M. Morphogenetic furrow initiation and progression during eye development in Drosophila: the roles of decapentaplegic, hedgehog and eyes absent. Development. 2000;127: 1325–1336. Available: http://www.ncbi.nlm.nih.gov/pubmed/10683184 10683184 10.1242/dev.127.6.1325

[19] Pappu KS, Chen R, Middlebrooks BW, Woo C, Heberlein U, Mardon G. Mechanism of hedgehog signaling during Drosophila eye development. Development. 2003;130: 3053–62. Available: http://www.ncbi.nlm.nih.gov/pubmed/12756186 12756186 10.1242/dev.00534

[20] Lopes CS, Casares F. Hth maintains the pool of eye progenitors and its downregulation by Dpp and Hh couples retinal fate acquisition with cell cycle exit. Dev Biol. 2010;339: 78–88. 10.1016/j.ydbio.2009.12.020 20036228

[21] Firth LC, Baker NE. Retinal determination genes as targets and possible effectors of extracellular signals. Dev Biol. 2009;327: 366–375. 10.1016/j.ydbio.2008.12.021 19135045 PMC2650007

[22] Pai CY, Kuo TS, Jaw TJ, Kurant E, Chen CT, Bessarab DA, et al The Homothorax homeoprotein activates the nuclear localization of another homeoprotein, extradenticle, and suppresses eye development in Drosophila. Genes Dev. 1998;12: 435–46. Available: http://www.pubmedcentral.nih.gov/articlerender.fcgi?artid=316489&tool=pmcentrez&rendertype=abstract 9450936 10.1101/gad.12.3.435 PMC316489

[23] Pichaud F, Casares F. Homothorax and iroquois-C genes are required for the establishment of territories within the developing eye disc. Mech Dev. 2000;96: 15–25. 10.1016/S0925-4773(00)00372-5 10940621

[24] Vollmer J, Fried P, Sánchez-Aragón M, Lopes CS, Casares F, Iber D. A quantitative analysis of growth control in the Drosophila eye disc. Development. 2016; 10.1242/dev.129775 26965369

[25] Foty R a., Forgacs G, Pfleger CM, Steinberg MS. Liquid properties of embryonic tissues: Measurement of interfacial tensions. Phys Rev Lett. 1994;72: 2298–2301. 10.1103/PhysRevLett.72.2298 10055839

[26] Forgacs G. Surface tension and viscoelastic properties of embryonic tissues depend on the cytoskeleton. Biol Bull. 1998;194: 328–330. Available: http://www.ncbi.nlm.nih.gov/pubmed/11536875 11536875 10.2307/1543103

[27] Dillon R, Gadgil C, Othmer HG. Short- and long-range effects of Sonic hedgehog in limb development. Proc Natl Acad Sci U S A. 2003;100: 10152–10157. 10.1073/pnas.1830500100 12930894 PMC193531

[28] Zhou S, Lo WC, Suhalim JL, Digman M a., Gratton E, Nie Q, et al Free extracellular diffusion creates the Dpp morphogen gradient of the Drosophila wing disc. Curr Biol. 2012;22: 668–675. 10.1016/j.cub.2012.02.065 22445299 PMC3338872

[29] Kicheva A, Pantazis P, Bollenbach T, Kalaidzidis Y, Bittig T, Jülicher F, et al Kinetics of Morphogen Gradient Formation. Science (80-). 2007;315: 521–525. 10.1126/science.1135774 17255514

[30] Fried P, Iber D. Dynamic scaling of morphogen gradients on growing domains. Nat Commun. 2014;5: 5077 10.1038/ncomms6077 25295831

[31] Geva-Zatorsky N, Issaeva I, Mayo A, Cohen A, Dekel E, Danon T, et al Using bleach-chase to measure protein half-lives in living cells. Nat Protoc. 2012;7: 801–11. 10.1038/nprot.2012.028 22461069

[32] Torroja C, Gorfinkiel N, Guerrero I. Patched controls the Hedgehog gradient by endocytosis in a dynamin-dependent manner, but this internalization does not play a major role in signal transduction. Development. 2004;131: 2395–2408. 10.1242/dev.01102 15102702

[33] Panáková D, Sprong H, Marois E, Thiele C, Eaton S. Lipoprotein particles are required for Hedgehog and Wingless signalling. Nature. 2005;435: 58–65. 10.1038/nature03504 15875013

[34] Kicheva A, Bollenbach T, Wartlick O, Jülicher F, Gonzalez-Gaitan M. Investigating the principles of morphogen gradient formation: From tissues to cells. Curr Opin Genet Dev. 2012;22: 527–532. 10.1016/j.gde.2012.08.004 22959150

[35] Nahmad M, Stathopoulos A. Dynamic Interpretation of Hedgehog Signaling in the Drosophila Wing Disc. Scott MP, editor. PLoS Biol. Public Library of Science; 2009;7: 15 Available: http://www.ncbi.nlm.nih.gov/pubmed/19787036 10.1371/journal.pbio.1000202 PMC2744877 19787036

[36] Aguilar-Hidalgo D, Domínguez-Cejudo MA, Amore G, Brockmann A, Lemos MC, Córdoba A, et al A Hh-driven gene network controls specification, pattern and size of the Drosophila simple eyes. Development. 2013;140: 82–92. 10.1242/dev.082172 23154412

[37] Masucci JD, Miltenberger RJ, Hoffmann FM. Pattern-specific expression of the Drosophila decapentaplegic gene in imaginal disks is regulated by 3’ cis-regulatory elements. Genes Dev. 1990;4: 2011–2023. 10.1101/gad.4.11.2011 2177439

[38] Treisman JE, Rubin GM. wingless inhibits morphogenetic furrow movement in the Drosophila eye disc. Development. 1995;121: 3519–3527. Available: http://www.ncbi.nlm.nih.gov/pubmed/8582266 8582266 10.1242/dev.121.11.3519

[39] Rogers EM, Brennan CA, Mortimer NT, Cook S, Morris AR, Moses K. Pointed regulates an eye-specific transcriptional enhancer in the Drosophila hedgehog gene, which is required for the movement of the morphogenetic furrow. Development. 2005;132: 4833–43. 10.1242/dev.02061 16207753

[40] Domínguez M, Hafen E. Hedgehog directly controls initiation and propagation of retinal differentiation in the Drosophila eye. Genes Dev. 1997;11: 3254–64. Available: http://www.pubmedcentral.nih.gov/articlerender.fcgi?artid=316756&tool=pmcentrez&rendertype=abstract 9389656 10.1101/gad.11.23.3254 PMC316756

[41] Borod ER, Heberlein U. Mutual regulation of decapentaplegic and hedgehog during the initiation of differentiation in the Drosophila retina. Dev Biol. 1998;197: 187–197. 10.1006/dbio.1998.8888 9630745

[42] Corrigall D, Walther RF, Rodriguez L, Fichelson P, Pichaud F. Hedgehog signaling is a principal inducer of Myosin-II-driven cell ingression in Drosophila epithelia. Dev Cell. 2007;13: 730–42. 10.1016/j.devcel.2007.09.015 17981140

[43] Escudero LM, Bischoff M, Freeman M. Myosin II regulates complex cellular arrangement and epithelial architecture in Drosophila. Dev Cell. 2007;13: 717–29. 10.1016/j.devcel.2007.09.002 17981139

[44] Schlichting K, Dahmann C. Hedgehog and Dpp signaling induce cadherin Cad86C expression in the morphogenetic furrow during Drosophila eye development. Mech Dev. 2008;125: 712–28. 10.1016/j.mod.2008.04.005 18539010

[45] Rieckhof GE, Casares F, Ryoo HD, Abu-Shaar M, Mann RS. Nuclear translocation of extradenticle requires homothorax, which encodes an extradenticle-related homeodomain protein. Cell. 1997;91: 171–83. Available: http://www.ncbi.nlm.nih.gov/pubmed/9346235 9346235 10.1016/s0092-8674(00)80400-6

[46] Moreno E, Basler K, Morata G. Cells compete for decapentaplegic survival factor to prevent apoptosis in Drosophila wing development. Nature. 2002;416: 755–9. 10.1038/416755a 11961558

[47] Burke R, Basler K. Dpp receptors are autonomously required for cell proliferation in the entire developing Drosophila wing. Development. 1996;122: 2261–9. Available: http://www.ncbi.nlm.nih.gov/pubmed/8681806 8681806 10.1242/dev.122.7.2261

[48] Harmansa S, Hamaratoglu F, Affolter M, Caussinus E. Dpp spreading is required for medial but not for lateral wing disc growth. Nature. 2015;527: 317–22. 10.1038/nature15712 26550827

[49] Hamaratoglu F, de Lachapelle AM, Pyrowolakis G, Bergmann S, Affolter M. Dpp signaling activity requires Pentagone to scale with tissue size in the growing Drosophila wing imaginal disc. PLoS Biol. 2011;9: e1001182 10.1371/journal.pbio.1001182 22039350 PMC3201923

[50] Akiyama T, Gibson MC. Decapentaplegic and growth control in the developing Drosophila wing. Nature. 2015;527: 375–8. 10.1038/nature15730 26550824

[51] Averbukh I, Ben-Zvi D, Mishra S, Barkai N. Scaling morphogen gradients during tissue growth by a cell division rule. Development. 2014;141: 2150–2156. 10.1242/dev.107011 24803660

[52] Ryder E, Spriggs H, Drummond E, St Johnston D, Russell S. The Flannotator—a gene and protein expression annotation tool for Drosophila melanogaster. Bioinformatics. 2009;25: 548–9. 10.1093/bioinformatics/btp012 19126575

[53] Callejo A, Culi J, Guerrero I. Patched, the receptor of Hedgehog, is a lipoprotein receptor. Proc Natl Acad Sci U S A. 2008;105: 912–7. 10.1073/pnas.0705603105 18198278 PMC2242683

[54] Martín FA, Morata G. Compartments and the control of growth in the Drosophila wing imaginal disc. Development. 2006;133: 4421–6. 10.1242/dev.02618 17035294

[55] Casares F, Mann RS. Control of antennal versus leg development in Drosophila. Nature. 1998;392: 723–6. 10.1038/33706 9565034

[56] Hastie T, Tibshirani R. Generalized Additive Models. Stat Sci. 1986;1: 297–310. 10.1214/ss/1177013604 8548102

[57] Eden E, Geva-Zatorsky N, Issaeva I, Cohen A, Dekel E, Danon T, et al Proteome half-life dynamics in living human cells. Science. 2011;331: 764–8. 10.1126/science.1199784 21233346

[58] Kang M, Day CA, Kenworthy AK, DiBenedetto E. Simplified equation to extract diffusion coefficients from confocal FRAP data. Traffic. 2012;13: 1589–600. 10.1111/tra.12008 22984916 PMC3731631

