# Supplementary figures and images for: A Model of the Spatio-temporal Dynamics of Drosophila Eye Disc Development
Source: PLoS Comput Biol. 2016 Sep 14;12(9):e1005052. doi: 10.1371/journal.pcbi.1005052 (PMC5023109; doi:10.1371/journal.pcbi.1005052)

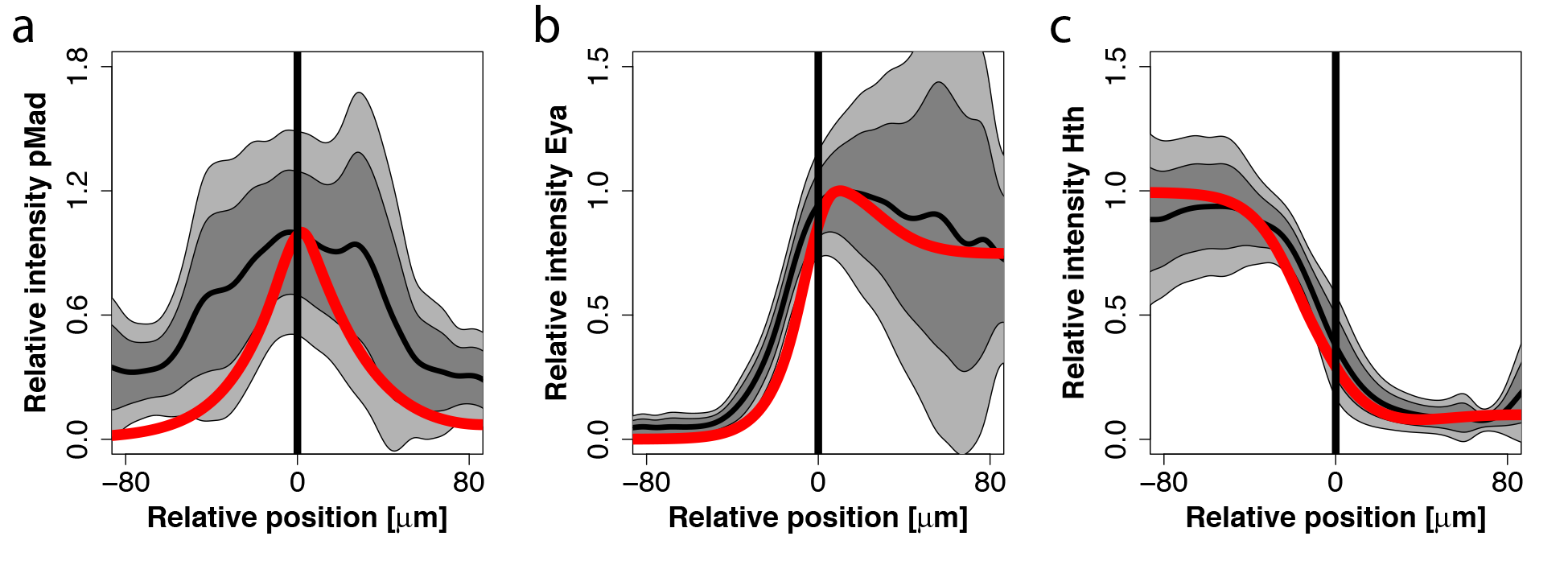

Supplement: S1 Fig — (a-c) Comparison of measured pMad (a), Hth (b) and Eya (c) profiles to the simulated gradients at 25h. Black lines indicate the estimated mean concentration over 60 bins created from the experimental data, dark grey areas indicate the estimated standard error and light grey areas indicate the 90% confidence interval for pMad, Hth, and Eya. (TIF) [file pcbi.1005052.s001.tif]
