# Supplementary material for: A Model of the Spatio-temporal Dynamics of Drosophila Eye Disc Development
Source: PLoS Comput Biol. 2016 Sep 14;12(9):e1005052. doi: 10.1371/journal.pcbi.1005052 (PMC5023109; doi:10.1371/journal.pcbi.1005052)
Supplement: S1 Table — The table summarizes all parameter values used in the model along with the evidence. All components i have the same production rate pi. (DOCX) [file pcbi.1005052.s002.docx]

| **Type** | **Parameter** | **Simulation Value** | **Reference Value** | **References** |
| --- | --- | --- | --- | --- |
| **Size** | $L_{AP}(0)$ | 74 μm | Extrapolation from measured data |  |
|  | $L_{DV}(0)$ | 172 μm | Extrapolation from measured data |  |
|  | $A_{0}$ | 10000 μm^2^ | Extrapolation from measured data | [24] |
|  | $k_{0}$ | 4 × 10^-5^ s^-1^ | Fitted growth kinetics | [24] |
|  | $\delta_{PL}$ | 0.0107 μm^-1^ | Fitted growth kinetics | [24] |
| **Diffusion** | $D_{Dpp}$ | 0.1 μm^2^ s^-1^ | Wing Disc value | [29] |
|  | $D_{Hh}$ | 0.033 μm^2^ s^-1^ | Measured | Fig. 3 |
|  | $D_{Hth}$ | 0.00025 μm^2^ s^-1^ | ~0, acts inside cell |  |
|  | $D_{Eya}$ | 0.00025 μm^2^ s^-1^ | ~0, acts inside cell |  |
|  | $D_{pMad}$ | 0.00025 μm^2^ s^-1^ | ~0, acts inside cell |  |
| **Degradation** | $\delta_{Dpp}$ | 2.5 × 10^-4^ s^-1^ | Half-life of Dpp in wing disc | [29] |
|  | $\delta_{Hh}$ | 1 × 10^-3^ s^-1^ | Characteristic length in wing disc | [29,34] |
|  | $\delta_{Hth}$ | 7 × 10^-5^ s^-1^ | Measured | Fig. 2 |
|  | $\delta_{Eya}$ | 1 × 10^-3^ s^-1^ | Assuming fast kinetics |  |
|  | $\delta_{pMad}$ | 1 × 10^-3^ s^-1^ | Assuming fast kinetics |  |
| **Prod & Regulation** | $p_{i}$ | 7 × 10^-5^ s^-1^ | Non-Dimensional concentration |  |
|  | $\eta$ | 3 × 10^-10^ μm^-1^ | Non-Dimensional concentration |  |
|  | $\bar{K}_{pMad}$ | 0.0015 | Fitted concentration profile | Fig. 7 |
|  | $\bar{K}_{Hh}$ | 0.01 | Fitted concentration profile | Fig. 7 |
|  | $K_{Dpp}$ | 0.003 | Fitted concentration profile | Fig. 7 |
|  | $K_{Hh}$ | 0.008 | Fitted concentration profile | Fig. 7 |
|  | $K_{pMad}$ | 0.02 | Fitted concentration profile | Fig. 7 |
|  | $n_{Dpp}$ | 1 | Fitted concentration profile | Fig. 7 |
|  | $n_{Hh}$ | 1 | Fitted concentration profile | Fig. 7 |
|  | $n_{pMad}$ | 2 | Fitted concentration profile | Fig. 7 |
|  | $\theta_{Hh}$ | 0.0465 | Fitted concentration profile | Fig. 7 |
|  | $\theta_{Hth}$ | 0.375 | Fitted concentration profile | Fig. 7 |
